# Fever, Seizures, and Basal Ganglia Lesions

**DOI:** 10.1177/00099228231194856

**Published:** 2023-08-15

**Authors:** Lakshmimathy Subramanian, Rohan Rakheja, Kirk Leifso, Anupam Sehgal, Nadine Morrison-Levy

**Affiliations:** 1 Department of Pediatrics, Kingston Health Sciences Centre, Queen’s University, Kingston, ON, Canada

**Educational Objectives**

- To understand the heterogenous and rare neurological manifestation of Parvovirus B19
- To broaden the differential for the neurological presentation of encephalitis and encephalopathy in pediatric patients

## Case Report

We describe the case of a previously healthy 17-year-old boy admitted to the hospital after a generalized tonic-clonic seizure that followed a 3-day history of fever, malaise, and headache.

At presentation, he was in no distress with a heart rate of 75 beats per minute, respiratory rate of 14 breaths per minute, temperature of 36.5 °C, oxygen saturation on room air at 99%, and blood pressure of 127/ 82 mm Hg. His Glasgow Coma Score (GCS) was 13, with disorientation to time and place. His receptive language was intact. However, he had difficulty finding words and responded with one-word answers. He had both long and short-term memory loss but no focal neurological signs. Pupils were equal and reactive to light, with normal range of eye movement and no nystagmus. Fundoscopy was normal. There was no facial asymmetry or tongue fasciculations. He had normal tone, muscle bulk, power 5/5, and reflexes 2+ in the upper and lower limbs. Sensation, gait, and coordination were intact. There were no abnormal movements or meningeal signs. Brudzinski and Kernig signs were not elicited. A complete cardiorespiratory and gastrointestinal exam was unremarkable. There was no rash, joint tenderness or swelling.

Investigations at presentation showed high white blood cell count of 11.8 × 10^9^/ L, neutrophils at 7.7 × 10^9^/ L, lymphocytes at 2.9 × 10^9^/ L, hemoglobin of 146 g/L, and platelet of 194 × 10^9^/ L. Cerebrospinal fluid (CSF) cell count was elevated at 54 × 10^6^/L, protein was high at 0.64 g/L, and glucose was 2.8 mmol/L. Repeat labs revealed mild anemia on day 4 of admission (lowest hemoglobin 134 g/L, hematocrit 0.385), which resolved without interventions by day 10 of admission. There was no thrombocytopenia. A complete septic workup was completed, including a nasopharyngeal swab for viral testing, blood and urine cultures, and a lumbar puncture. Bacterial and urine cultures demonstrated no growth. There were no organisms seen on the CSF gram stain and culture. Blood serum and CSF serology were negative for commonly encountered bacterial and viral infections. A broad differential was considered, but all tests were negative, including the autoimmune and metabolic panel (Table 1).

**Table 1.** List of Investigations Completed to Screen for Various Etiologies.

Target	Test	Source	Result
**Microbiology**			
Parvovirus B19	Antibodies	Blood serum and CSF	Serology IgM negative Serology IgG positive Serology DNA positive CSF IgM negative CSF IgG negative CSF DNA negative
* Escherichia coli* K1, Haemophilus influenzae, Listeria monocytogenes, Neisseria meningitidis, *Streptococcus agalactiae*, *Streptococcus pneumoniae*, Human herpesvirus 6	Antibodies	CSF	Negative
Coronavirus 19	Polymerase chain reaction (PCR) (real-time DNA)	Nasopharyngeal swab	Negative
Influenza A and B	PCR RNA	Nasopharyngeal swab	Negative
Respiratory syncytial virus (RSV)	PCR RNA	Nasopharyngeal swab	Negative
Mycoplasma	PCR DNA	Nasopharyngeal swab	Negative
Chlamydia pneumoniae	PCR DNA	Nasopharyngeal swab	Negative
Methicillin-resistant *Staphylococcus aureus* (MRSA)	PCR culture	Nasopharyngeal swab	Negative
Cytomegalovirus (CMV)	Antibodies	Blood serum	IgG positive (past exposure) IgM negative
Powassan virus	Antibodies	Blood serum	IgM negative (<1:10)
West Nile virus	Antibodies	Blood serum	IgM negative
Flavivirus	Antibodies	Blood serum	IgG negative
Human immunodeficiency virus (HIV) 1 and 2	P24 antigen and antibodies	Blood serum	Negative
Borrelia burgdorferi (Lyme)	Antibodies	Blood serum	IgG negative IgM negative
Toxoplasma gondii	Antibodies	Blood serum	IgG negative
Bartonella henselae	Antibodies	Blood serum	IgG negative (<1:64)
Enterovirus	PCR RNA	CSF	Negative
Parechovirus	PCR RNA	CSF	Negative
Epstein-Barr virus (EBV)	PCR DNA	CSF	Negative
EBV	Capsid and nuclear IgG	Blood serum	Negative
Herpes simplex virus (HSV) 1 and 2	PCR DNA	CSF	Negative
Varicella-zoster virus (VZV)	PCR DNA	CSF	Negative
**Autoimmune**			
NMDA receptor	Screen	CSF	Negative
Oligoclonal banding	Screen	CSF	Negative
Autoimmune encephalitis panel	Screen	CSF Blood serum	Negative
Neuromyelitis panel	Screen	CSF	Negative
Wilson’s disease (copper and ceruloplasmin level)	Screen	Blood serum	Negative
ANA/ENA level	Screen	Blood serum	Negative
Rheumatoid factor	Screen	Blood serum	Negative (<15)
DNA antibodies	Screen	Blood serum	Negative
p and c ANCA level	Screen	Blood serum	Negative
Extractible antibodies (dsDNA, Chromatin, Ribosomal Protein, SS-A 52, SS-A 60, SS-B, Sm, SmRNP, RNP A, RNP 68, Scl-70, Jo-1, and Centromere B)	Screen	Blood serum	Negative
**Metabolic**			
Amino acids profile	Screen	Urine and plasma	Negative
Organic acids	Screen	Urine	Negative
Lactate	Screen	Blood serum	Negative
Ammonia	Screen	Blood serum	Negative

Abbreviations: CSF, cerebrospinal fluid; DNA, deoxyribonucleic acid; PCR, polymerase chain reaction; RNA, ribonucleic acid; RSV, respiratory syncytial virus; MRSA, methicillin resistant staphylococcus aureus; CMV, cytomegalovirus; HIV, human immunodeficiency virus; EBV, Epstein-Barr virus; HSV, herpes simplex virus; VZV, varicella-zoster virus; NMDA, N-methyl-D-aspartate; ANA, antinuclear antibody; ENA, extractable nuclear antigen; ANCA, antineutrophil cytoplasmic antibody; SS, Sjogren’s Syndrome; RNP, ribonucleoprotein.

An electroencephalogram (EEG) done on admission was abnormal and had features of encephalopathy. Initial magnetic resonance imaging (MRI) brain (diffusion and susceptibility weighted imaging, T1, T2 post-gadolinium sequence) showed bilateral basal ganglia lesions on day 2 of admission. A follow-up MRI brain on day 5 showed progression involving the left parietal lobe and both frontal lobes. An MRI of the spine was normal. Given the patient’s history of consumption of illicit substances, a thorough investigation was pursued. However, urine toxicology and serum volatile substances screen were negative.

Since his encephalopathy worsened with increasing agitation and confusion on day 4 of admission, an expanded viral panel was completed on blood serum and cerebrospinal fluid (CSF) (Table 1). The Cytomegalovirus (CMV) IgG was positive, indicat-ing past infection. Serum parvovirus B19 (PVB19) DNA and antiB19-IgG were also positive. The serum antiB19-IgM, CSF DNA, and CSF antibodies (IgG and IgM) were negative (Table 1).

## Final Diagnosis

After extensive investigations, a diagnosis of parainfectious or postinfectious PVB19 encephalitis was confirmed in the context of PVB19 DNA detected in the serum with positive serum antiB19-IgG. This was supported by a negative repeat serum PVB19 DNA 4 weeks later.

## Hospital Course

The patient was empirically started on anti-viral (acyclovir 800 mg every 8 hours) and anti-seizure (levetiracetam 750 mg in the morning and 500 mg in the evening) medications when he presented to the hospital.

He received 5-day course of high-dose pulsed steroids, starting on day 6 of admission, resulting in significant clinical and radiological improvement. A repeat MRI brain on day 16 of the admission showed reduced edema. The patient steadily returned to his baseline within a week postdischarge, and the anti-epileptic was discontinued 6 months later. A repeat MRI brain at 6 months postdischarge was normal, and a repeat serology 4 weeks after the onset of illness was negative for serum PVB19 DNA.

## Discussion

Neurological complications arising from PVB19 infection are rare.^1^ Thus, there is a paucity of data in literature on PVB19 and neurological manifestations, especially in the pediatric population. Monteiro et al.^2^ identified 50 cases of children with neurological manifestations of PVB19 in their systematic review and case series analysis. Among those, encephalitis and encephalopathy were the most common and comprised about 38.8% of the total reported PVB19 cases.^2,3^ Douvoyianni et al.^3^ observed more neurological manifestations of PVB19 in children compared with adults. Some specific forms of encephalopathy, such as chorea encephalopathy, mild encephalitis/encephalopathy with reversible splenial lesion (MERS), and posterior reversible encephalopathy syndrome (PRES), were more frequently seen in children.^3,4^ There has been at least one case reported with acute autonomic sensory and motor neuropathy.^1^

In the systematic review by Barah et al.,^5^ rash was observed in only 33% of the children with parvovirus B19 neurological disease, while anemia and arthralgia were detected in 43.6% and 7.7% of the cases, respectively.

A study by Watanabe et al.^6^ reviewed PVB19 associated Pediatric neurological cases and identified impaired consciousness, seizures, and focal neurologic signs as the main presenting features. About 82.6% of those cases had anti-B19 serum IgM, and 90% had CSF DNA for PVB19.^6^ Thus, suggesting that serum IgM and CSF DNA are helpful tools for diagnosis.^6^

Our patient had altered mental status and seizures but no focal neurological signs. Interestingly, his serum was positive only for PVB19 DNA and anti-B19 IgG but not IgM. His CSF was negative for both DNA and antibodies. Consistent with our patient, Tonnellier et al.^7^ reported a case of a 28-year-old immunocompetent patient with PVB19 encephalitis whose CSF was negative for PVB19 DNA. This finding is supported by the theory that the central nervous system (CNS) lesions in PVB19 are more of an immune-mediated response to PVB19 infection than direct toxicity by the virus on the cerebral tissues.^1,4,6-8^ With a significant improvement in MRI brain imaging and clinical status following high-dose steroids, our case supports the immunomodulatory and inflammatory effects of PVB19 on the CNS.^7^

What was unusual in our case was that the patient developed serum antiB19-IgG but not IgM. Usually, IgM develops approximately 10 days after exposure and remains positive for a few months.^3,9^ IgG develops a few days after IgM and remains positive for life.^3,9^ There is a possibility of a false-positive and false-negative result as the source and type of viral antigen can affect the sensitivity of the laboratory test.^3,9,10^ Also, IgG could have been passively transferred from blood products, but our patient did not receive any intravenous immunoglobulin (IVIG).^9^ Since repeat serum polymerase chain reaction (PCR) was negative for PVB19 DNA, our diagnosis of parainfectious or postinfectious PVB19 encephalitis was confirmed. There was no other infectious etiology identified, particularly after extensive testing.

The MRI brain findings commonly seen in PVB19 infection are high-intensity signals in the basal ganglia, white matter and cerebellum.^4^ Additional hyperintense areas may also be found in the splenial part of the corpus callous.^4^ Similarly, our patient had basal ganglia lesions, which initially prompted our healthcare team to investigate toxic and metabolic causes due to the symmetrical involvement of the region.^11^ The basal ganglia, an active metabolic and vascular center, tend to be bilaterally affected by systemic causes. This includes toxic poisoning (carbon monoxide, methanol, and cyanide), metabolic abnormalities (hyperglycemia or hypoglycemia), vascular abnormalities (venous infarction or arterial occlusion), inflammatory conditions (neuro Behcet’s disease), liver disorders, and viral (flavivirus encephalitis, Influenza A acute necrotizing encephalopathy) and parasitic infections (Toxoplasmosis).^11^ These conditions were ruled out in our patient with extensive screening investigations (Table 1). Neurodegenerative disorders such as iron accumulation, Creutzfeldt-Jakob disease or Fahr disease that affect basal ganglia typically present with progressive disease.^11^ Our patient did not have the characteristic signs of MRI brain imaging or neurodegenerative signs of dystonia, bowel or bladder dysfunction or dysarthria.^11^ Nonetheless, since there is no presenting syndrome, defined classification criteria or MRI brain findings specific to PVB19 encephalitis, it is challenging to differentiate it from other etiologies and to make a clear diagnosis.^8,11^ Moreover, a case of PVB19 encephalitis has been reported with normal MRI brain findings.^1^

Systematic review and case series analysis in immunocompetent children with neurological manifestations of PVB19 by Monteiro et al.^2^ have not found any objective assessment of the efficacy of IVIG and/ or steroids for therapy. This has been consistent with other reviews by Barah et al.^5^ and Watanabe et al.^6^ However, several cases have noted clinical improvement in children with PVB19-associated neurological symptoms.^5^ Although the exact mechanism of action remains unknown, IVIG and steroid therapy have been suggested for severe cases in the absence of other effective treatments.^2,5,6^ Anti-seizure medications were used for cases with PVB19-associated seizures.^2^

## Conclusion

PVB19 should be considered as a cause in pediatric patients presenting with acute encephalitis, and this is supported by other studies.^1,4^ We also recommend considering PVB19 infection in pediatric patients with nonspecific bilateral CNS lesions, particularly involving the basal ganglia.

## Author Contributions

LS, RR, KL, AS and NM contributed to the conception and design of the report, analysis and interpretation of the case. LS and NM drafted the initial manuscript. KL, LS and NM critically revised the manuscript. All authors reviewed, provided final approval and agree to all aspects of work integrity and accuracy.
